# Emergent materials and industry 4.0 contribution toward pandemic diseases such as COVID-19

**DOI:** 10.1007/s42247-020-00102-4

**Published:** 2020-05-06

**Authors:** Mariam AlAli AlMaadeed

**Affiliations:** Qatar University, Doha, Qatar

Raw materials have played significant role in all industrial revolutions. Iron, steel, advanced machinery, cutting-edge tools, and structured factories have instigated the first industrial revolution in the UK. At its core, the production of steel has paved the way for the industry to flourish and built up the foundations for new era world and globalization. Owing to its strength, ease in designing, and reliability, steel proved to be an extremely useful utility in healthcare. Improvements in manufacturing and better standardization have resulted in the occurrence of second industrial revolution in the late nineteenth century up to the early twentieth century. Electricity, petroleum, and rubber-based materials were developed during this period, with wide applications in the medical and health fields. This was accompanied by regulations and standardizations which improved health care infrastructure and infection control systems. During that period, economic interdependence was evolving, and socio-economic systems around the world were being transformed. Globalization continued to grow exponentially throughout the third and fourth industrial revolutions, and technology played a major role in developing society and fulfilling global needs. The uniqueness of the fourth industrial revolution (Industry 4.0) is not to be solely attributed to the advancement in manufacturing and digital systems, but also to the convenience of transportation between geological borders and the relaxation of national boundaries.

Although the effects of globalization have been positive in most respects, this has arguably not been the case when it comes to global pandemics. Disease outbreaks have occurred several times during the past few decades, showcasing rapid evolution and spread. Despite the progress in medicine, globalization has raised other risks and consequences in disease outbreaks which can undermine public health systems, economies, and overall social behavior. With these socio-economic transformations and the nature of genetically modified strains of viruses, the outbreaks may become more frequent and lethal. Therefore, governments around the world should be well prepared for possible health crises.

In December 2019, the World Health Organization (WHO) announced the outbreak of an emergent infectious disease due to a specific strain of corona virus called SARS-CoV-2. The novel coronavirus disease (COVID-19) was declared by the WHO as a pandemic on March 11, 2020. According to WHO (https://www.who.int/emergencies/diseases/novel-coronavirus-2019), updated on April 9, 2020, there is an overall of 1,395,136 confirmed cases and total deaths of 81,580 worldwide.

Along with the efforts of the WHO, nation states should be ready to manage these infectious diseases by independently performing continuous monitoring, diagnostics, and prevention procedures (detect, prevent, and treat). The question of the moment is that what roles can industry 4.0 and its emergent materials can play in mitigating the harms of disease outbreaks as a whole, and COVID-19 in particular. Emergent materials are playing a central role in improving the health systems and in facing the consequences of the ongoing pandemic.

The detection of diseases is done through specific biomarkers in saliva, blood, or tissue samples. New devices based on nanomaterials can perform fast, inexpensive, and sensitive detections. Biosensors are common in the detection process. These sensors can be customized using new tailored nanomaterials for improved electrochemical and optical sensors. Such materials are used for their customized properties, specifically their conductivity, mechanical stability, and easy processing techniques. Some of the prime examples include quantum dots, nanofibers, nanowires, magnetic beads, metal-based nanoparticles, and nanocomposites.

The application of these materials in infectious disease analytical tests requires improvement in sensitivity, lowering both the cost and complexity process. Collaboration between researchers in materials science and industry can lead to a new effective way in improving the detection of epidemics through the reduction of the needed workflow and speedup the diagnostic procedures.

Building smaller and smarter testing systems is highly required as it will be easier to test the samples on the site where possible patients are expected. Emergent materials with nanotechnology are contributing through reducing the size of these systems. The application of emergent tailored nanomaterials will lead to usage of less quantities of the needed samples and chemicals used for testing, meanwhile also making the over-all process more practical.

Emergent materials can also play a role in antiviral activities. Well-known examples of such materials include graphene oxide and reduced graphene oxide. Their antiviral activities are attributed to their structure (single layer) and to the presence of a negative charge.

Moreover, emergent materials within Industry 4.0 are also contributing in supporting fatigued health systems during the current pandemic. For instance, some materials are improving the robotic systems and sensor abilities for sterilization and the detection of crowded areas. This is done through improving sensing materials which eventually lead to increase in the battery life. Additionally, the 3D printing of different types of polymers and composites is now a well-established technique providing much needed medical parts and custom components which are in short supply. Recent and present examples of such materials include face masks, shields, ventilator parts, etc.

Further research in different types of emergent materials and nanomaterials can be tested and tailored for better protection, analysis, and curing of pandemic diseases, including COVID-19. The Emergent Materials journal supports the development of materials in improving human welfare worldwide and welcomes contributions in advancing healthcare and technology systems with advanced emerging materials to combat and prevent disease outbreaks.

